# Facilitators and barriers to adherence to medical recommendations among adolescents with cancer: A systematic review

**DOI:** 10.1177/13674935231208502

**Published:** 2023-10-21

**Authors:** Ágata Salvador, Shivani Atul Mansuklal, Maria Moura, Carla Crespo, Luísa Barros

**Affiliations:** 1 HEI-Lab, 70887 Lusófona University, Lisbon, Portugal; 2 Research Center for Psychological Science, Faculty of Psychology, 37838 University of Lisbon, Lisbon, Portugal; 3 37810 Instituto Português de Oncologia de Lisboa Francisco Gentil EPE, Lisbon, Portugal

**Keywords:** Adherence, adolescent, cancer, compliance, determinants

## Abstract

We aimed to systematically review barriers/facilitators of adherence among adolescents with cancer (aged 10–24 years), following a comprehensive approach to adherence that goes beyond medication-taking. Empirical studies published in English exploring determinants of adherence to medical recommendations among adolescents with cancer were identified in MEDLINE, PsycInfo, and Web of Science, up to October 2021. Records and full-text articles were reviewed by two independent reviewers, and results were classified according to the World Health Organization’s (WHO) multidimensional adherence model. Eighteen studies were included. Despite heterogeneity in the definition and measurement of adherence, literature supported barriers/facilitators at patient, treatment, condition, healthcare team/system, and social/economic levels. Specifically, patient-related factors (i.e., psychological functioning and beliefs about disease and treatment) and social-related factors (i.e., family functioning) were major determinants of adolescent adherence. Few studies were conducted, and inconsistent findings were displayed for other dimensions (i.e., healthcare team/system, treatment, and condition-related factors). Adherence is a complex and multidetermined phenomenon. More research is needed to provide critical insights for policymakers and healthcare professionals in planning practices and interventions that effectively address meaningful barriers/facilitators of adolescents’ adherence.

## Introduction

Improvements in treatment and survival among paediatric cancer populations (Couzin-Frankel, 2019) have raised awareness of the required lifelong healthcare. In chronic diseases like cancer, patients are actively involved in their conditions’ management, being responsible for taking medication, following up with medical team, and engaging in health-promoting activities (Hinds and Linder, 2020). With increased availability of self-administered medications (Kennard et al., 2004; McGrady and Pai, 2019) and the shift to ambulatory settings (McGrady and Pai, 2019), patient’s adherence to medical regimens is a topic of increasing concern (McGrady and Pai, 2019).

Initially, compliance was used to describe the extent to which the patient passively follows medical advice, a definition that implies a paternalistic perspective (Aronson, 2007). Recent and contemporary literature has favoured the term adherence (Modi et al., 2020), which recognises the patient’s active involvement in treatment (Vandermorris et al., 2019). According to the World Health Organization (WHO, 2003), adherence refers to ‘the extent to which a person’s behaviour – taking medication, following a diet, and/or executing lifestyle changes – corresponds with agreed recommendations from a health care provider’ (p. 13). In light of this comprehensive definition, adherence is not limited to taking medication, comprising the avoidance of health-risk behaviours (substance use and sun exposure) as recommended by medical team (Vandermorris et al., 2019).

Cancer treatment requires a wide variety of health-related behaviours beyond taking medication (Vandermorris et al., 2019), namely, attending appointments, seeking medical help for urgencies, managing symptoms, undertaking preventive measures against infections, and engaging in a health-promoting lifestyle (physical activity, diet, avoiding smoking, and sun protection (Kodryn et al., 2009; Vandermorris et al., 2019). Despite the multidimensional nature of adherence, research has focused on medication-taking, largely overlooking other behaviours (Leader and Raanani, 2014).

Suboptimal adherence is a major concern in oncology (Verbrugghe et al., 2013), especially in paediatric context, with adolescents reporting higher rates of nonadherence than children (Lancaster et al., 1997; Tebbi et al., 1986) and adults (McGrady and Pai, 2019). Estimates suggest that up to 60% of adolescents (either wholly or as part of a sample with children or young adults) fail to adhere to their oral treatments (McGrady and Pai, 2019; Pai and McGrady, 2015; Ruddy et al., 2009).

Nonadherence poses significant adverse consequences for patients, families, and healthcare systems; concretely poor prognosis; increased risk of relapse and mortality; overuse of services; and higher healthcare costs (Butow et al., 2010; Smith and Shuchman, 2005; Taddeo et al., 2008; WHO, 2003). These consequences highlight the critical need to explore factors driving nonadherence, to better recognise those at risk and to inform better practices to promote adherence.

WHO (2003) has proposed a model of adherence to long-term therapies that recognises five categories of factors: patient/caregiver (e.g., knowledge and beliefs about the disease), treatment (e.g., length and complexity of the treatment regimen and side effects), condition (e.g., disease severity and level of physical disability), healthcare team and system (e.g., patient-provider relationship), and social/economic (e.g., socioeconomic status and ethnicity). This model has been subsequently applied to paediatric oncology context (Goh et al., 2016).

Scholars have also advocated the value of exploring adherence through a developmentally oriented standpoint (Butow et al., 2010; Vandermorris et al., 2019), considering the specific challenges that may arise depending on the developmental period. A chronic health condition during adolescence may pose challenges to developmental tasks, which in turn may amplify barriers of treatment adherence (Butow et al., 2010; Leader and Raanani, 2014). Concretely, cancer treatment may result in increased dependence on parents (Vandermorris et al., 2019), lack of peer interaction, plans suspension, and body image and identity concerns (Butow et al., 2010). As such, nonadherence may be a way of gaining control and responsibility for one’s life (Landier, 2011; Leader and Raanani, 2014; Taddeo et al., 2008), being accepted by and socialising with peers, and may express difficulties in integrating a health condition into an evolving sense of self (Taddeo et al., 2008).

Despite cognitive maturation, adolescents often report difficulties in conceiving actions’ long-term and unseen consequences (Landier, 2011; Taddeo et al., 2008) and impulse control (Vandermorris et al., 2019), which may explain health-risk behaviours (alcohol and/or drug use) and low treatment adherence (Taddeo et al., 2008). Thus, an empirical focus on adolescent patients is pivotal because of this group's unique biological and psychosocial characteristics, which differ from those faced by younger children or adults (Alderman et al., 2019).

Reviews addressing nonadherence among adolescents with cancer have been published in the last decades, offering valuable overviews of the available empirical evidence. However, most have included studies focused on a single segment of adherence behaviours (taking medication; McGrady and Pai, 2019; Pai and McGrady, 2015) and combined different age cohorts (adolescents, children, and/or young adults, Goh et al., 2016; McGrady and Pai, 2019).

It must be noted the variability in the definition of adolescence age range. Following a recent biopsychosocial perspective, adolescence corresponds to the period from 10 to 24 years, beginning with puberty and ending with role transitions to adulthood (Sawyer et al., 2018). To the best of our knowledge, only one review has applied this age criterion and included literature up until 2008 (Taddeo et al., 2008). Additionally, it has not presented a clear definition of adherence and the range of behaviours in its scope.

To date, reviews aiming to identify adherence-related factors have not simultaneously applied a comprehensive definition of adherence and a biopsychosocial perspective of adolescence.

### Aim

The aim is to identify determinants (barriers/facilitators) of adherence among adolescents with cancer, following a comprehensive approach to adherence (broad range of adherence behaviours, as opposed to a medication-taking only) and a contemporary definition of adolescence (10–24 years).

## Method

### Search strategy

This review was conducted according to the Preferred Reporting Items for Systematic Reviews and Meta-Analyses (PRISMA) guidelines (Page et al., 2021). Systematic search on three selected databases – MEDLINE, PsycInfo, and Web of Science – was conducted in November 2021, using a combination of highly sensitive medical subject headings (MeSH) terms (see supplementary material - Table S1). Previous searches were conducted by the first two authors to identify relevant terms. Studies were included if: written in English; peer-reviewed; empirical studies presenting original data (no reviews, case reports, commentaries, books, practice guidelines, conference abstracts, and dissertations); included adolescents between 10 and 24 years old (following a contemporary and broad age definition that encompasses the biological, social, and neurocognitive development of adolescence) with any cancer diagnosis; and examined one or more determinants of adherence to medical recommendations. Studies were excluded if focused on providers’ adherence behaviours and included any other disease than cancer. No limits on publication date were applied. Considering the dearth of literature addressing the topic, studies using either qualitative or quantitative methodologies were included. Studies with multiple age cohorts (adolescents and children or adolescents and adults) were included only if adherence and its determinants could be uniquely tied to the adolescent population.

Results were exported to EndNote (Clarivate, v20). After duplicate removal, titles and abstracts were reviewed by AS and SA independently (91.3% inter-rater agreement). Then, a similar process was performed for full-text screening, with discrepancies about eligibility solved through discussion. After full-text screening, a hand search of the reference lists of included studies and relevant reviews was conducted to identify additional relevant studies.

### Data extraction and quality appraisal

A form was developed to extract information from the included studies (Table 1). Data extraction was primarily performed by AS and reviewed by SA. Additionally, AS categorised the identified determinants, following a combination of deductive (for predefined WHO’s five dimensions) and inductive approaches (for categories under major dimensions). The list of categories was then revised by the remaining authors.

**Table 1.** Summary of data extracted from included studies.

Authors and date	Country	Type of study	Sample	Age	Diagnosis	Adherence focus and measure	Barriers/facilitators identified
Dolgin et al., 1986	USA	CS	*N* = 65; 27 females and 38 males	12–18 (14.2)	Mixed	Physician report on gross non-compliance (missing appointments, treatment resistance or refusal, treatment discontinuation, and non-compliance with follow-up requirements)	- Treatment side effects
							- Poor family and social support
							- Denial of the illness severity
							- Lack of belief in treatment efficacy
Heneghan et al., 2020	USA	CS	*N* = 15; 3 females and 12 males	16–19 (17)	Acute lymphoblastic leukaemia	Self- and parent-reports on oral medication adherence	**Psychological capability to:** find out what medications are; what the medication does; the side effects; remember to take medication; tracker if had taken the medication; remember appointments; the doctor’s instructions about how to take medication; and to refill medication
							**Physical capability to:** get the adolescent to take medication
							**Physical opportunity to:** pick up medication; to get to appointments; to contact doctor; the adolescent is away/at school; and access to health records
							**Social opportunity to:** meet other youths with the same condition; contact community organisations; and meet other parents of adolescents with cancer
							**Reflective motivation to:** find out what the medication does and the side effects of medication
Hollen and Hobbie, 1996	UK	CS	*N* = 52; 27 females and 25 males	14–19	Mixed	Self-report of risk behaviours (smoking, alcohol, and drug use)	- Poor-quality decision-making for important choices
							- Types of treatment (with/without cognitive impairment) – NS
Hullmann et al., 2015	USA	CS	*N* = 103; 45 females and 58 males	13–19 (15.77 +/− 1.77)	Mixed	Self- and parent-reports on medication adherence	**Individual factors**
							*Demographic variables*
							- Gender, age, and race/ethnicity – NS
							*Disease and treatment variables*
							- Days spent in outpatient clinic
							- Diagnosis, time since diagnosis, days spent inpatient, treatment intensity, and treatment modality – NS
							*Psychosocial variables*
							- Fewer future-oriented goals
							- Self-efficacy and affect – NS
							**Family factors**
							- Less family support
							- Less overprotectiveness from secondary caregiver
							- Income, parent education, parent marital status, perceived caring from primary and secondary caregivers, primary caregivers’ overprotection, and family functioning – NS
							**Community factors**
							- Social support from friends – NS
							**Reasons for not taking medication:** forget, being away from home, difficulty in swallow, taste of medication, not feeling well, unpleasant side-effects, interference with activity, believing that it is unnecessary, refuse/defiant, medication ran out/didn’t fill, and inability to afford
Kennard et al., 2004	USA	CS	*N* = 44; 17 females and 27 males	13–17 (15.3 +/− 1.8)	Mixed	Drug assay, self- and parent-reports on medication adherence	**Nonadherence measured by assay**
							- Depression
							- Family incongruence
							- Low self-concept
							- Age, gender, socioeconomic status, duration of illness, and psychosocial adjustment – NS
							**Nonadherence based on self-report**
							- Low self-concept
							- Depression, psychosocial adjustment, and family incongruence – NS
							**Nonadherence based on parent-report**
							- Low psychosocial adjustment
							- Depression, self-concept, and family incongruence – NS
Kondryn et al., 2009	USA	CS	*N* = 33; 14 females and 19 males	16–24 (20.1 +/− 3.7)	Mixed	Self-report on low-risk (with no immediate consequences) and high-risk (with immediate consequences) nonadherence behaviours	**Low-risk nonadherence**
							- Considering stopping treatment
							**High-risk nonadherence**
							- Considering stopping treatment – NS
Malbasa et al., 2007	USA	QS	*N* = 6; 4 females and 2 males	16–23 (19.17)	Acute lymphoblastic leukaemia	Adherence to oral mediation explored in focus groups interviews	**Factors mentioned as explaining suboptimal adherence**
							- Need for normalcy with peers (treatment make them different from their peers), school and extracurricular activities (treatment is a barrier to participation in activities), and treatment and disease course (transition from intensive to maintenance treatment is a barrier to normalcy)
							- Egocentrism
							- Regression to concrete thinking
							- Lack of parental support, supervision, and encouragement
Mancini et al., 2012	France	QS	*N* = 12; 5 females and 7 males	11–17	Acute lymphoblastic leukaemia	Interviews to explore medication adherence	**Factors mentioned as risk factors for forgetfulness**
							- Feeling of recovery/cured
							- Aversion toward the treatment
							- Lack of parental supervision
							- Distractions from social life
							- Fatigue
							- Scheduling therapy for bedtime
McGrady et al., 2018	USA	CS	*N* = 20; 8 females and 12 males	15.35–23.71 (18.66 +/−2.95)	Mixed	Self-report to explore adherence to medication	**Factors that motivate adherence**
							- Perceived positive impact of medication adherence on long-term goals
							- Perceived medication effectiveness
							- Parent/significant other support and encouragement
							- Perceived of low impact of medication on daily activities
Psihogios et al., 2021	USA	CS	*N* = 18; 4 females and 14 males	15–22 (17.94 +/− 2.31)	Acute lymphoblastic leukaemia	Electronic monitoring, self- and physician-reports of medication adherence	**Predictors of adherence measured through electronic device:**
							- Weekend (vs. weekdays)
							- Low motivation
							- Worse negative affect
							- Difficulties with talking to parents about thoughts and feelings
							- Pain, fatigue, nausea, positive affect, disagreement with parent, loneliness, being at home, and being with family – NS
Psihogios et al., 2020	USA	MM	**Study 1** *N* = 72 36 females and 36 males **Study 2** *N* = 116 females and 5 males	**Study 1** (16.9 +/2212 2.5) **Study 2** (17.1 +/– 2.7)	Mixed	**Study 1 (CS)** Parent-report on medication, diet, and physical activity adherence **Study 2 (QS)** Interview to explore medication, diet, and physical activity adherence	**Study 1**
							*Top barriers of adherence*
							- Medication: forgetfulness, not feeling well, being away from home, and believing it’s not necessary
							- Diet: not feeling well, no appetite, and like/don’t like certain foods
							-Physical activity: not feeling well, having no time, and low motivation
							*Imperfect (vs. perfect) medication adherent*
							- Higher number of medication barriers
							- More depressive symptoms
							- Worse quality of life
							- Worse family functioning
							- Worse perceptions of family condition management efficacy
							- More family financial stress
							- Higher out-of-pocket medical expenses
							*Imperfect (vs. perfect) diet adherent*
							- Higher number of diet barriers
							*Imperfect (vs. perfect) physical activity adherent*
							- Worse family functioning
							**Study 2**
							**Factors mentioned as barriers/facilitators to adherence**
							*Physical*: Physical symptoms/side effects and instability of symptoms as barriers
							*Psychological*: Low mood, lack of peer interaction, fear of weight gain, and inability to recognise long-term benefits of adherence as barriers
							*Family*: family readjustment to allocate treatment, positive family communication, family cohesion, multiple family members involvement in regimen, caregiver worry as facilitators; caregiver involvement may be a facilitator (i.e., promotes adherence in the short-term) and a barrier (i.e., can lead to disagreements, conflict, and impacts on adolescents’ autonomy);
							*Access to care*: insurance that does not considered the preferred medication formulation, out-of-pocket expenses, logistical difficulties (e.g., transportation, pharmacy, and coordination of work and clinic visits), complexity of treatment (i.e., requiring more remote support), and complexity of health system (i.e., multiple providers, treatments, and insurance authorisations) as barriers
Tebbi et al., 1989	USA	CS	*N* = 20; 10 females and 10 males	10–23 (15.0)	—	Self- and parent-reports on medication adherence	- Attributions of responsibility for the cause and solution of cancer (medical vs compensatory model) – NS
							- Parent/child concordance regarding attributional perspective – NS
Tercyak et al., 2005	USA	CS	*N* = 75; 39 females and 36 males	11–21 (14.2 +/− 2.4)	Mixed	Self-report on risk behaviours (i.e., smoking; physical activity and sun protection)	- Older patients
							- Greater symptoms of depression in the context of greater parent–child conflict
							- Gender, race, cancer type, and time since diagnosis – NS
Weaver et al., 2016	USA	QS	*N* = 40; 16 females and 24 males	12–18.6 (15.5 +/− 2.2)	Mixed	Interviews to explore medical instructions	**Factors mentioned as facilitators to adherence**
							- Professional partnerships and care relationships
							- Gratitude for medical care
Wicks et al., 2010	New Zealand	QS	*N* = 10; 4 females and 6 males	16–11 (19)	Mixed	Interviews to explore adherence to medication	**Factors mentioned as barriers to adherence**
							- Feeling of ‘loss of control’
Yagci-Kupeli et al., 2017	Turkey	RS	*N* = 293; 104 females and 189 males	15–19	Mixed	Medical files to assess non-compliance to treatment (i.e., irregular attendance and refusing therapy)	- Diagnosis: patients with non-Hodgkin lymphoma and osteosarcoma were shown to be more non-compliant
							- Gender and early stage or metastatic disease – NS
Jamison et al., 1986	USA	CS	*N*=27; 16 females and 11 males	12–18, (15.5 +/− .32)	Mixed	Providers-report on adolescents cooperation (i.e., compliance with cancer treatment)	- Age (older adolescents were less cooperative)
							- Poor self-image
							- Low perception of cancer as a serious disease
							- External locus of control (powerful others)
							- Sex, socioeconomic status, number of people at home, and knowledge of cancer – NS
Blotcky et al., 1985	USA	CS	*N* = 20	**Refusers:** 13–17 (14.9)	Mixed	Self-reports on treatment refusal	**Refusers (vs. consenters)**
							- Lower state anxiety
							- Higher trait anxiety
							- Lower subjective distress
							- Higher external locus of control
							- Higher religiosity
							- Family satisfaction, hopelessness, satisfaction with physician, mother’s coping, and mother’s anxiety – NS
							**Reasons for refusing treatment**
							- Fear of appearance
							- Fear of peer’s reactions
							- Fear of side effects
							- Fear of painful medical procedures

*Note*: CS = cross-sectional; LS = longitudinal study; RS = retrospective study; QS = qualitative study; MM = mixed-method; NS = non-significant.

Quality assessment of the included studies was performed independently by AS and SA, and results were later discussed. Given that this review included both quantitative and qualitative designs, we used an adaptation of CASP quality assessment tool (Critical Appraisal Skills Programme, 2018), previously used by Nielson and colleagues (2021). Based on the lack of research in the field, risk of bias was not used as a reason for exclusion.

## Results

### Study characteristics

**Figure 1.**
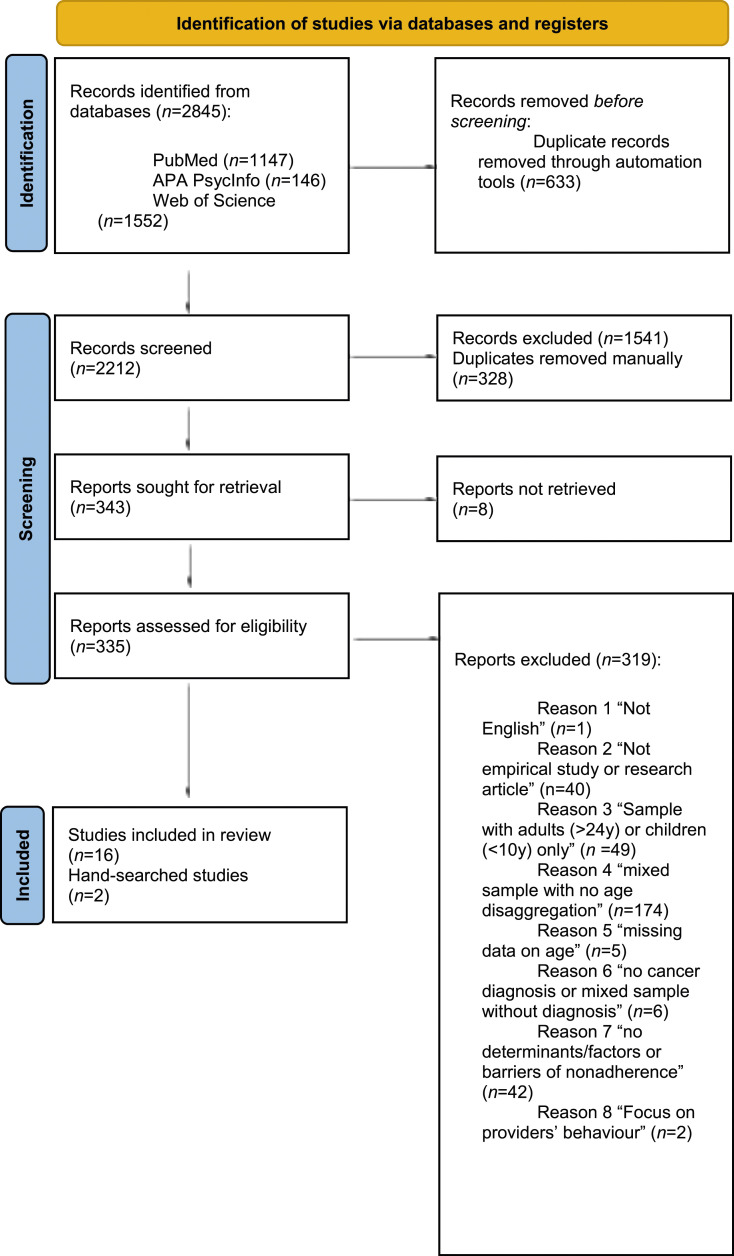
PRISMA 2020 flow diagram of the study selection process.

Eighteen eligible studies were included (Figure 1). Table 1 summarises studies’ characteristics. Published between 1985 and 2021, most were conducted in the United States of America (USA; *n* = 14; 77.8%). The majority were quantitative (*n* = 13; 72.2%). From these, 12 (66.7%) were cross-sectional, and one (5.6%) used a retrospective design. Four studies (22.2%) were qualitative, and one (5.6%) applied mixed-methods. The sample sizes ranged from 6 to 293 participants and most of the studies included mixed cancer diagnoses (*n* = 13; 72.2%).

Nine studies (50%) focused on medication adherence only (Heneghan et al., 2020; Hullmann et al., 2015; Kennard et al., 2004; Malbasa et al., 2007; Mancini et al., 2012; McGrady et al., 2018; Psihogios et al., 2021; Tebbi et al., 1989; Wicks and Mitchel, 2010), and eight (44.4%) followed a comprehensive definition of adherence, including other behaviours related to treatment (Dolgin et al., 1986; Jamison et al., 1986; Kondryn et al., 2009; Weaver et al., 2016; Yağcı-Küpeli et al., 2017) or general health-related behaviours (physical activity, diet, smoking, and sun protection; Hollen and Hobbie, 1996; Psihogios et al., 2020; Tercyak et al., 2005). One study (5.6%) focused on treatment refusal (Blotcky et al., 1985).

Regarding the adherence measurement, objective (drug assays, electronic monitoring, and medical files) and subjective methods (self, parent, and physician reports and interviews) were used. Most of the studies relied on a single method (*n* = 12; 66.7%): five (27.8%) used self-reports (Blotcky et al., 1985; Hollen and Hobbie, 1996; Kondryn et al., 2009; McGrady et al., 2018; Tercyak et al., 2005), two (11.1%) used physicians reports (Dolgin et al., 1986; Psihogios et al., 2021), one (5.6%) relied on medical files (Yağcı-Küpeli et al., 2017), and four (22.2%) conducted qualitative interviews (Malbasa et al., 2007; Mancini et al., 2012; Weaver et al., 2016; Wicks and Mitchel, 2010). Six studies (33.3%) combined multiple sources and types of assessment: three (16.7%) combined self and parental reports (Heneghan et al., 2020; Hullmann et al., 2015; Tebbi et al., 1989); one (5.6%) applied parent-report and in-person interviews (Psihogios et al., 2021); and two (11.1%) used three types of measures (Kennard et al., 2004; Psihogios et al., 2021).

### Study quality

Quality assessment results are displayed in Table 2. Although the majority of quantitative studies were cross-sectional, most have not suggested causal inferences. The main concern refers to small samples, recruited in a single institution. Despite the appropriate data collection methods, the validity of questionnaires needed to be established in some cases. Generally, data analysis has been well described, although most did not control for confounders. Concerning qualitative studies, few discussed data saturation, and the relationship between the researcher and participants.

**Table 2.** Critical appraisal skills programme quality of evidence.

Authors and date	Clear aim	Appropriate methodology	Appropriate design	Appropriate sampling and recruitment	Appropriate data collection	Consideration of research/participant relationship	Consideration of ethical issues	Rigorous analysis	Findings clear stated	Is the research valuable
Dolgin et al., 1986	P	Y	P	P	Y	N/A	N	P	P	P
Heneghan et al., 2020	Y	Y	Y	P	Y	N/A	Y	Y	Y	Y
Hollen and Hobbie, 1996	Y	Y	Y	P	Y	N/A	Y	Y	Y	Y
Hullman et al., 2015	Y	Y	Y	Y	Y	N/A	Y	Y	Y	Y
Kennard et al., 2004	Y	Y	Y	P	Y	N/A	Y	Y	Y	Y
Kondryn et al., 2009	Y	Y	Y	P	Y	N/A	Y	Y	Y	Y
Malbasa et al., 2007	Y	Y	Y	Y	P	N	Y	Y	Y	Y
Mancini et al., 2012	Y	Y	Y	P	Y	N	Y	N	N	P
McGrady et al., 2018	Y	Y	Y	P	Y	N/A	Y	Y	Y	Y
Psihogios et al., 2021	Y	Y	P	P	Y	N/A	Y	Y	Y	Y
Psihogios et al., 2020	Y	Y	P	P	P	N	Y	Y	Y	Y
Tebbi et al., 1989	Y	Y	Y	N	P	N/A	N	Y	Y	P
Tercyak et al., 2005	Y	Y	Y	P	Y	N/A	Y	Y	Y	Y
Weaver et al., 2016	Y	Y	Y	Y	Y	Y	Y	Y	Y	Y
Wicks et al., 2010	Y	Y	Y	Y	P	N	Y	Y	Y	Y
Yagci-Kupeli et al., 2017	P	Y	Y	P	Y	N/A	N	Y	P	P
Jamison et al., 1986	Y	Y	P	P	P	N/A	N	Y	Y	P
Blotcky et al., 1985	Y	Y	Y	P	P	N/A	Y	Y	Y	Y

*Note*: Y = yes (fully met criterion), P = partially met criterion, N = no (criterion not met), N/A = not applicable.

### Review of facilitators/barriers to adherence

Based on WHO model of adherence (2003), facilitators/barriers to adherence were identified and divided into five categories. Figure S1 synthesises the results (see Supplementary Material).

#### Patient/caregiver-related facilitators/barriers (*n* = 16; 88.9%)

Three factors were identified for patient-related category: patients’ psychological functioning; cognitive functioning; and disease/treatment-related beliefs, with 16 studies supporting these factors. First, studies (*n* = 9; 50%) found that adolescents reporting worse psychosocial functioning, concretely worse quality of life (Psihogios et al., 2020), higher levels of trait anxiety (Blotcky et al., 1985), and depressive symptoms (Kennard et al., 2004; Psihogios et al., 2020) were less likely to adhere. Depressive symptoms seemed to be especially problematic for health-risk behaviors under the conditions of parent–child conflict (Tercyak et al., 2005). Other factors adversely impacting adherence included lack of perceived control (Blotcky et al., 1985; Jamison et al., 1986; Psihogios et al., 2020; Wicks and Mitchel, 2010), poor self-concept (Kennard et al., 2004), self-image (Blotcky et al., 1985), and lack of motivation and worse affect (Psihogios et al., 2020, 2021). Defiance was also reported as a reason for medication nonadherence (Hullmann et al., 2015). In these circumstances, parents reported difficulties in getting the adolescent to take the medication (Heneghan et al., 2020).

Concerning cognitive functioning, concrete thinking (Malbasa et al., 2007; Psihogios et al., 2020), egocentrism (Malbasa et al., 2007), and forgetfulness (Heneghan et al., 2020; Hullmann et al., 2015; Psihogios et al., 2020) were identified as impacting adherence. Patients reporting medication nonadherence described difficulties remembering to take medication and medical instructions (Heneghan et al., 2020). Studies have also mentioned inability to recognise non-adherence long-term consequences (Malbasa et al., 2007; Psihogios et al., 2020) and lower future-oriented goals among nonadherent patients (Hullmann et al., 2015). Poor decision-making style regarding lifestyle and health matters (not searching choices; weighing the pros and cons of consequences) was also associated with health-risk behaviours (smoking and alcohol; Hollen and Hobbie, 1996).

Regarding patients’ disease/treatment-related beliefs, denial of illness severity (Dolgin et al., 1986; Jamison et al., 1986) and lack of perceived need (Hullmann et al., 2015; Mancini et al., 2012; Psihogios et al., 2020; ) and treatment efficacy (Dolgin et al., 1986; McGrady et al., 2018) were related to low adherence. Also, adolescents reported poor adherence when perceiving that treatment strongly impacts normalcy in everyday activities (McGrady et al., 2018), school (Malbasa et al., 2007), peer relationships (Malbasa et al., 2007), and long-term goals (McGrady et al., 2018). Fear of painful procedures (Blotcky et al., 1985) and of potential side effects (Blotcky et al., 1985), like weight gain (Psihogios et al., 2020) and appearance modifications (Blotcky et al., 1985) were reported by nonadherent adolescents. Lack of knowledge about medication was also a barrier to medication adherence (Heneghan et al., 2020). When assessing low- (with no immediate effects) versus high-risk nonadherent behaviours (with immediate consequences) and their relationship to adolescents’ attitudes towards treatment, it was found that patients who had considered stopping treatment reported significantly higher low-risk behaviours but not high-risk behaviours (Kondryn et al., 2009).

#### Treatment-related facilitators/barriers (*n* = 4; 22.2%)

Four studies supporting two treatment-related factors were identified: treatment side effects and complexity of treatment regimen. First, treatment side-effects (fatigue, nausea, and pain) were identified as a reason for nonadherence (Dolgin et al., 1986; Hullmann et al., 2015; Mancini et al., 2012; Psihogios et al., 2020). Regarding treatment complexity, nonadherence risk increased when treatment regimen required more remote support (Psihogios et al., 2020), outpatient clinic visits, interfered more with activity, and required difficulty swallowing pill-form medications (Hullmann et al., 2015).

#### Condition-related facilitator/barriers (*n*=3; 16.7%)

Three studies supported the condition-related factors: type of diagnosis and disease course. First, only one study found that adolescents with non-Hodgkin lymphoma and osteosarcoma (vs other cancer types) were more likely to irregularly attend to medical appointments and refuse therapy (Yağcı-Küpeli et al., 2017). Additionally, a study found that the transition from intensive to maintenance treatment was associated with medication nonadherence risk (Malbasa et al., 2007; Mancini et al., 2012).

#### Healthcare team/system-related facilitators/barriers (*n*=3; 16.7%)

Two factors were found: communication and support from the healthcare team; and access to medication and health insurance, with three supporting studies. First, whereas good patient–provider relationships (partnership and caring relationships) facilitate adherence (Weaver et al., 2016), difficulties in contacting medical staff and understanding medical instructions act as barriers (Heneghan et al., 2020).

Second, inadequate access to medication and health insurance was shown to hinder adherence, namely, problems in medication distribution (Heneghan et al., 2020; Psihogios et al., 2020) and inadequate health insurance plans (Psihogios et al., 2020).

#### Social and economic facilitators/barriers (*n* = 12; 66.7%)

Four social and economic factors were found: financial/logistical difficulties; sociodemographic variables; family; and social interactions. Twelve studies supported these factors. Among financial/logistical difficulties, family financial stress, the perception of out-of-pocket expenses (Psihogios et al., 2020), and difficulties in affording medication (Hullmann et al., 2015 were associated with poor adherence. Other barriers correspond to logistical issues, namely, transport problems (Heneghan et al., 2020; Psihogios et al., 2020), caregivers’ difficulties in coordinating job and clinic visits (Psihogios et al., 2020), and difficulties in integrating medical recommendations in routines (Heneghan et al., 2020; Hullmann et al., 2015; Mancini et al., 2012; Psihogios et al., 2020).

Second, regarding sociodemographic features, only age showed a significant association with adherence, with two studies suggesting that older adolescents tend to be less adherent to general medical treatment procedures and health-related behaviors (Jamison et al., 1986; Tercyak et al., 2005).

Third, studies suggested that family functioning may play a role in adherence. While family cohesion and positive communication were reported as facilitators (Psihogios et al., 2020, 2021), worse family functioning, family difficulties in managing condition/treatment (Psihogios et al., 2020), and parent–child incongruence in describing family environment (Kennard et al., 2004) may negatively impact adherence.

Another group of family factors pertains to the support provided by family members (Dolgin et al., 1986; Hullmann et al., 2015) and their involvement in treatment which act as facilitators (Psihogios et al., 2020). Specifically, parents’ emotional support, supervision, and encouragement were found to promote adolescent medication adherence (Malbasa et al., 2007; Mancini et al., 2012; McGrady et al., 2018). A study added that parental involvement promoted adherence to medical recommendations in the short-term, but could lead to conflict and hinder patients' autonomy, thus acting as a barrier in the long-term (Psihogios et al., 2020). Finally, nonperfect adherent adolescents to medication tend to perceive their secondary caregiver as less overprotective (Hullmann et al., 2015).

Fourth, mixed findings are apparent for social interactions. While some studies found that social support and interactions act as facilitators (Dolgin et al., 1986; Heneghan et al., 2020; Psihogios et al., 2020), another study revealed that social life might distract adolescents from their medication regimen (Mancini et al., 2012). Additionally, those who refused treatment mentioned fear of peers' reaction as a reason for not consenting treatment (Blotcky et al., 1985). Finally, and concerning the caregivers’ social life, the lack of social opportunities to meet other parents of adolescents with cancer and organisations were associated with medication nonadherence (Heneghan et al., 2020).

## Discussion

This review identified 18 studies examining barriers/facilitators of adherence to medical recommendations among adolescents with cancer. There is evidence that adherence among this population is influenced by an interplay of factors on the five WHO dimensions (2003), with patient- and social factors being the major determinants of adherence.

First, adolescents with cancer were less likely to adhere when reporting poor psychological functioning (Jamison et al., 1986; Kennard et al., 2004; Psihogios et al., 2020). Psychopathological symptoms are especially linked with low adherence when adolescents are simultaneously dealing with a worse family environment (Tercyak et al., 2005). This supports the exponential negative impact of co-occurrence of risk factors (Rutter, 1979). Moreover, there is evidence that when adolescents are undergoing treatment, psychological functioning may explain their adherence to medication but not to health-promoting behaviors (Psihogios et al., 2020). Thus, depending on the disease phase, barriers may influence different facets of adherence.

Patient’s psychological functioning has been extensively explored in the literature through the assessment of a wide variety of dimensions (e.g., depression, self-efficacy, and affect), and some studies found no evidence for a significant association with adolescents’ adherence (e.g., Hullmann et al., 2015). Thus, it is conceivable that different psychological dimensions may impact adherence behaviors differently.

Research also indicated that nonadherence seems to be explained by inadequate patient’s disease/treatment perceptions, namely, perception of condition as not severe and treatment as not effective (Dolgin et al., 1986; Hullmann et al., 2015; McGrady et al., 2018; Psihogios et al., 2020).

Considering the role of patients’ cognitive functioning, the immediacy of the behaviours’ consequences is a factor to be considered, as adolescents’ difficulties in recognising their actions’ long-term consequences were associated with nonadherence (Malbasa et al., 2007; Psihogios et al., 2020).

Regarding social/economic-related determinants, family variables were major factors linked to adherence. While those factors pertaining to family functioning (assessed by family communication, cohesion, and conflict) showed some inconsistencies in the pattern of associations with adherence (with some studies showing no evidence for a significant association, e.g., Psihogios et al., 2021), support and involvement from family members in treatment regimen were consistently identified as playing a critical role. Specifically, parental support and supervision may facilitate adolescents’ adherence (Malbasa et al., 2007; Mancini et al., 2012; McGrady et al., 2018), especially in the short-term (Psihogios et al., 2020). In addition to the role of primary caregivers, research highlights that the involvement of secondary caregivers may act as a key facilitator of adherence to medication-taking (Hullmann et al., 2015).

Mixed findings were apparent regarding the role of social interactions, possibly indicating that peer relationships may act in multiple ways during adolescence. While quantitative studies generally document the positive role of peer interactions (Heneghan et al., 2020; Psihogios et al., 2020), qualitative research showed that adolescents also recognise challenging dynamics that may act as a barrier (Mancini et al., 2012).

Still in social/economic domain, nonadherence risk is linked with financial difficulties (Psihogios et al., 2020), especially when assessed by subjective reports (vs objective measures like income). Logistical difficulties (with transport, coordinating medical visits, and family routines; Psihogios et al., 2020; Heneghan et al., 2020), in turn, have consistently been identified as posing significant barriers to adherence to medical recommendations. Together these findings highlight the relevance of considering the family's subjective experience of difficulties in accomplishing the treatment regimen and responsibilities in their routines.

Among sociodemographic factors, older adolescents tend to report poorer adherence to general medical procedures and health-related behaviors (Jamison et al., 1986; Tercyak et al., 2005), with no differences in medication-taking (Hullmann et al., 2015; Kennard et al., 2004). This result supports the relevance of a developmental stance to understand adherence among adolescents. It is conceivable that adherence behaviors are more prevalent during early and middle adolescence because younger adolescents are more dependent on their caregivers’ supervision. On the other hand, during late adolescence, individuals gain autonomy from their caregivers (Vandermorris et al., 2019). This developmental task may explain a higher rate of nonadherence to general behaviors that do not have a clear or direct relationship with medical regimen.

Concerning treatment/condition characteristics, no clear relationships have been found across the few studies addressing these factors. While some findings supported that adverse treatment side effects (Mancini et al., 2012; Psihogios et al., 2020, 2021) and complex treatment regimens (Hullmann et al., 2015; Psihogios et al., 2020) may present barriers to adherence, others found no evidence for significant associations (Hullmann et al., 2015; Psihogios et al., 2021).

Regarding type of diagnosis, only one study found evidence of a significant effect (Yağcı-Küpeli et al., 2017). This result must be interpreted considering that authors collected data from medical files from 1970 to 2008. Medical advances have been registered, possibly explaining the inconsistencies with results from recent studies, which found no evidence of an association between diagnosis and adherence (Hullmann et al., 2015; Tercyak et al., 2005).

For disease course, a qualitative study found that the ambiguity of transitioning from intensive to maintenance treatment is perceived as a barrier (Malbasa et al., 2007): patients feel recovered with no evidence of disease but must continue therapy (Malbasa et al., 2007; Mancini et al., 2012). Rather than the objective nature of the disease/treatment itself (diagnosis, time since diagnosis, severity, and treatment intensity), adherence may be better explained by the adolescents' subjective perception of the disease/treatment and the challenges to be faced. Furthermore, adolescents may experience greater challenges and barriers to adherence in specific treatment phases, without necessarily reporting more non-adherent behaviors.

Vandermorris et al. (2017) argued that, compared to treatment/illness features, adolescent's relationship with healthcare system is a better determinant of adherence. Notably, the included studies have largely overlooked the role of the healthcare team and system-related factors, with few studies specifically exploring the topic. Studies suggest that collaborative and supportive interactions (Weaver et al., 2016) and good communication (Heneghan et al., 2020) may positively impact adherence. Additionally, inadequate access to medication and health insurance was found to be related to poor adherence (Heneghan et al., 2020; Psihogios et al., 2020).

### Limitations

This review has several limitations. Search process was limited to peer-reviewed studies published in English, which may have narrowed the number of studies and relevant findings may have been missed. Due to scarce literature specifically focused on adolescence and a comprehensive definition of adherence that goes beyond medication-taking, we included studies with small sample sizes and incomplete methodological relevant data. Additionally, limitations related to the inclusion of studies with different definitions and approaches to assessing adherence and barriers/facilitators must be considered, as it precludes the ability to draw robust conclusions about the effect of each factor on adherence. Finally, considering the delays in role transitions to adulthood in past decades, we followed a contemporary definition of adolescence proposed by Sawyer (2018). Nevertheless, this expanded definition may lead to the inclusion of heterogeneous samples regarding developmental challenges that may interfere with adherence.

### Recommendations for future research

This review highlights areas of improvement in future research. First, it is difficult to draw firm conclusions due to mixed findings that may reflect a wide variety of definitions and methods for measuring (non)adherence. Although most of the included studies focused on medication-taking only, a considerable number jointly addressed multiple behaviours. Such a broad perspective may hinder a deeper understanding of potential specificities of adherence phenomenon. In line with this, additional research specifically exploring relevant health behaviours like physical activity, smoking, alcohol use, or sun protection is needed (Wu et al., 2015). Similarly, future studies should address therapy refusal as a distinct form of nonadherence (Spinetta et al., 2002).

Concerning the adherence measurement, subjective reports have been used in most studies. Future research could apply objective methods combined with subjective reports, and multiple sources of information to strengthen the adherence assessment (Modi et al., 2020; Quittner et al., 2000; Vandermorris et al. 2019). This is particularly important, as one study showed that the pattern of associations with determinants varied according to the type of adherence measurement (Kennard et al., 2004). Another potential direction for future research is the use of qualitative designs, which would provide a deeper perspective regarding the underlying beliefs and the multiple meanings attached to the adolescents’ experience of adherence (Fiese and Bickman, 1998).

Most quantitative studies followed cross-sectional designs. Longitudinal work would not only ascertain the direction of effects but also facilitate the examination of mediation models to further explore the complex interplay between adherence determinants. Furthermore, repeated assessment following adolescents prospectively through the different phases could provide insights about which barriers/facilitators are more prevalent in each illness phase. It is also recommended that quantitative studies use larger sample sizes. Additionally, most studies relied on one-institution samples, leading to a lack of knowledge on specific context-related factors. Considering that previous research found low adherence among children from ethnic minorities (Bhatia et al., 2012), cultural and ethnic differences should be considered in the context of adherence among adolescents.

Finally, additional research is required to further elucidate how condition/treatment- and healthcare teams and system-related factors (e.g., alignment to the dimensions of patient and family-centred care approaches) influence adherence. A much needed emphasis in future research is dismantling which factors work as barriers/facilitators for whom and under which conditions. Clarifying this is critical for effective practices and intervention efforts that might improve response to treatment. Moreover, further research attention could be given to the role of caregiver’s functioning and peer social interactions in adolescent adherence. Lastly, given the focus of the included studies on barriers to adherence, encompassing a more competence/protection oriented-approach to identify ‘key’ facilitators may be one promising area for future research.

### Implications for practice

Framed in family-centred approaches, future directions may include routine screening practices to determine patients at the highest risk of nonadherence (monitoring potential difficulties/barriers; Coyne et al., 2019). In this regard, this review indicates that older adolescents, undergoing complex treatment regimens, with adverse treatment side effects, and transitioning from the intensive to the maintenance phase are at higher risk of poor adherence.

This review also identified modifiable factors, highlighting potential intervention targets. First, as previously suggested (Vandermorris et al., 2019), findings support the relevance of education-based strategies, to address adolescents’ beliefs about disease/treatment (promoting age-appropriate knowledge on disease and treatment necessity, treatment side-effects and medical instructions, and addressing patients’ treatment concerns). Likewise, strategies to support adolescents' striving for normalcy and forgetfulness may be key targets within an intervention.

Second, this review’s findings point to the relevance of psychosocial support-based interventions, specifically targeting psychological difficulties and relationship dynamics both at family and peer levels. Moreover, improving patient–provider interactions may also facilitate adherence among this population (Pritchard et al., 2006).

Finally, results also suggest that another beneficial path is addressing financial and logistical constraints by providing financial assistance (Coyne et al., 2019) and, when possible, actively involving patients and families in care decisions (Butow et al., 2010; Pyke-Grimm et al., 2020), so that the care needs do not collide with family routines. Furthermore, broader health changes promoting access to care needs (medication, health insurance) are known to be valuable for sustainable changes (Warnick et al., 2020).

## Conclusion

This review provides an updated synthesis of adherence determinants among adolescents with cancer, following a comprehensive approach to adherence and a contemporary definition of adolescence. Despite the small number of studies and a large amount of heterogeneity in the adherence definition and measurement, several potentially modifiable adherence barriers/facilitators were identified. Adherence to medical recommendations among adolescents with cancer is influenced by factors within each of the five domains of WHO (2003); patient, treatment, condition, healthcare system-related, and social/economic). Given the complex nature of adherence, there is room for further research on how multiple factors jointly influence the adolescents’ experience. Identifying the potential barriers/facilitators is critical for both policymakers and healthcare professionals in planning strategies that effectively address issues faced by adolescents with cancer.

## Supplemental Material

Supplemental Material - Facilitators and barriers to adherence to medical recommendations among adolescents with cancer: A systematic review Supplemental Material for Facilitators and barriers to adherence to medical recommendations among adolescents with cancer: A systematic review by Ágata Salvador, Shivani Atul Mansuklal, Maria Moura, Carla Crespo and Luísa Barros in Journal of Child Health Care.

## References

[bibr1-13674935231208502] Alderman EM, Breuber CC and Committee On Adolescence (2019) Unique needs of the adolescent. Pediatrics 144(6): e20193150. DOI: 10.1542/peds.2019-3150. 31740496

[bibr2-13674935231208502] Aronson JK (2007) Compliance, concordance, adherence. British Journal of Clinical Pharmacology 63(4): 383–384. DOI: 10.1111/j.1365-2125.2007.02893.x. 17378797 PMC2203247

[bibr3-13674935231208502] Bhatia S Landier W Shangguan M , et al. (2012) Nonadherence to oral mercaptopurine and risk of relapse in Hispanic and non-hispanic white children with acute lymphoblastic leukemia: a report from the children’s oncology group. Journal of Clinical Oncology 30(17): 2094–2101. DOI: 10.1200/JCO.2011.38.9924. 22564992 PMC3601449

[bibr4-13674935231208502] Blotcky AD Cohen DG Conatser C , et al. (1985) Psychosocial characteristics of adolescents who refuse cancer treatment. Journal of Consulting and Clinical Psychology 53(5): 729–731. DOI: 10.1037//0022-006x.53.5.729. 3863849

[bibr5-13674935231208502] Butow P Palmer S Pai A , et al. (2010) Review of adherence-related issues in adolescents and young adults with cancer. Journal of Clinical Oncology 28(32): 4800–4809. DOI: 10.1200/jco.2009.22.2802. 20212260

[bibr6-13674935231208502] Couzin-Frankel J (2019) Beyond survival. Science 363(6432): 1166–1169. DOI: 10.1126/science.363.6432.1166. 30872515

[bibr7-13674935231208502] Coyne KD Trimble KA Lloyd A , et al. (2019) Interventions to promote oral medication adherence in the pediatric chronic illness population: a systematic review from the children’s oncology group. Journal of Pediatric Hematology/Oncology Nursing 36(3): 219–235. DOI: 10.1177/1043454219835451. PMC6487845 30943831

[bibr8-13674935231208502] Critical Skills Appraisal Programme - Casp (2018) 10 questions to help you make sense of qualitative research. Available at: https://casp-uk.net/

[bibr9-13674935231208502] Dolgin MJ Katz ER Doctors SR , et al. (1986) Caregivers’ perceptions of medical compliance in adolescents with cancer. Journal of Adolescent Health Care 7(1): 22–27. DOI: 10.1016/S0197-0070(86)80090-0. 3943999

[bibr10-13674935231208502] Fiese BH Bickham NL (1998) Qualitative inquiry: an overview for pediatric psychology. Journal of Pediatric Psychology 23(2): 79–86. DOI: 10.1093/jpepsy/23.2.79. 9585634

[bibr11-13674935231208502] Goh XTW Tan YB Thirumoorthy T , et al. (2016) A systematic review of factors that influence treatment adherence in paediatric oncology patients. Journal of Clinical Pharmacy and Therapeutics 40(1): 1–7. DOI: 10.1111/jcpt.12441. 28045208

[bibr12-13674935231208502] Heneghan MB Hussain T Barrera L , et al. (2020) Applying the COM-B Model to Patient-Reported Barriers to Medication Adherence in Pediatric Acute Lymphoblastic Leukemia. Worcester, MA: Pediatric Blood & Cancer, e28216. DOI: 10.1002/pbc.28216. 32068338

[bibr13-13674935231208502] Hinds PS Linder L (2020) Pediatric Oncology Nursing: Defining Care through Science. New York, NY: Springer. DOI: 10.1007/978-3-030-25804-7.

[bibr14-13674935231208502] Hollen PJ Hobbie WL (1996) Decision making and risk behaviours of cancer-surviving adolescents and their peers. Journal of Pediatric Oncology Nursing 13(3): 121–134. DOI: 10.1177/104345429601300304. 8755441

[bibr15-13674935231208502] Hullmann SE Brumley LD Schwartz LA (2015) Medical and psychosocial associates of nonadherence in adolescents with cancer. Journal of Pediatric Oncology Nursing 32(2): 103–113. DOI: 10.1177/1043454214553707. 25366574 PMC4410359

[bibr16-13674935231208502] Jamison RN Lewis S Burish TG (1986) Cooperation with treatment in adolescent cancer patients. Journal of Adolescent Health Care 7: 162–167. DOI: 10.1016/s0197-0070(86)80032-8. 3700192

[bibr17-13674935231208502] Kennard BD Stewart SM Olvera R , et al. (2004) Nonadherence in adolescent oncology patients: preliminary data on psychological risk factors and relationships outcome. Journal of Clinical Psychology in Medical Settings 11(1): 31–39. DOI: 10.1023/B:JOCS.0000016267.21912.74.

[bibr18-13674935231208502] Kondryn HJ Edmondson CL Hill JW , et al. (2009) Treatment non-adherence in teenage and young adult cancer patients: a preliminary study of patient perceptions. Psycho-Oncology 18: 1327–1332. DOI: 10.1002/pon.1541. 19267369

[bibr19-13674935231208502] Lancaster D Lennard L Lilleyman JS (1997) Profile of non-compliance in lymphoblastic leukemia. Archives of Disease in Childhood 76: 365–366. DOI: 10.1136/adc.76.4.365. 9166035 PMC1717135

[bibr20-13674935231208502] Landier W (2011) Age span challenges: adherence in pediatric oncology. Seminars in Oncology Nursing 27(2): 142–153. DOI: 10.1016/j.soncn.2011.02.006. 21514483

[bibr21-13674935231208502] Leader A Raanani P (2014) Adherence-related issues in adolescents and young adults with hematological disorders. Acta Haematologica 132: 348–362. DOI: 10.1159/000360197. 25228561

[bibr22-13674935231208502] Malbasa T Kodish E Santacroce SJ (2007) Adolescent adherence to oral therapy for leukemia: a focus group study. Journal of Pediatric Oncology Nursing 24(3): 139–151. DOI: 10.1177/1043454206298695. 17475980

[bibr23-13674935231208502] Mancini J Simeoni M-C Parola N , et al. (2012) Adherence to leukemia maintenance therapy: a comparative study among children, adolescents, and adults. Pediatric Hematology & Oncology 29: 428–439. DOI: 10.3109/08880018.2012.693150. 22712832

[bibr24-13674935231208502] McGrady ME Pai AL (2019) A systematic review of rates, outcomes, and predictors of medication non-adherence among adolescents and young adults with cancer. Journal of Adolescent and Young Adult Oncology 8(5): 485–494. DOI: 10.1089/jayao.2018.0160. 31038372 PMC6791468

[bibr25-13674935231208502] McGrady ME Prosser LA Thompson AN , et al. (2018) Application of a discrete choice experiment to assess adherence-related motivation among adolescents and young adults with cancer. Journal of Pediatric Psychology 43(2): 172–184. DOI: 10.1093/jpepsy/jsx104. 29049671 PMC5896606

[bibr26-13674935231208502] Modi AC Gutierrez-Colina AM Driscoll KA (2020) Emerging areas. In: Modi AC Driscoll KA (eds) Adherence and Self-Management in Pediatric Populations. Cambridge, MA: Academic Press, pp. 409–423.

[bibr27-13674935231208502] Nielson S Bray L Carter B , et al. (2021) Physical restraint of children and adolescents in mental health inpatient services: a systematic review and narrative synthesis. Journal of Child Health Care 25(3): 342–367. DOI: 10.1177/1367493520937152. 32633554 PMC8422777

[bibr28-13674935231208502] Page MJ McKenzie JE Bossuyt PM , et al. (2021) The PRISMA 2020 statement: an updated guideline for reporting systematic reviews. BMJ 372(71). DOI: 10.1136/bmj.n71. PMC8005924 33782057

[bibr29-13674935231208502] Pai ALH McGrady ME (2015) Assessing medication adherence as a standard of care in pediatric oncology. Pediatric Blood and Cancer 62: s818–828. DOI: 10.1002/pbc.25795. 26700926

[bib9136749352314] Psihogios A. M. Li Y. Ahmed A. Huang J. Kersun L. S. Schwartz L. A. Barakat L. P. (2021) Daily text message assessments of 6-mercaptopurine adherence and its proximal contexts in adolescents and young adults with leukemia: A pilot study. Pediatric blood & cancer 68(2): e28767. Available at: 10.1002/pbc.28767. 33073479 PMC10313157

[bibr30-13674935231208502] Psihogios A, M Shwartz LA Ewing KB , et al. (2020) Adherence to multiple treatment recommendations in adolescents and young adults with cancer: a mixed methods multi-informant investigation. Journal of Adolescent and Young Adult Oncology 9(6): 651–661. DOI: 10.1089/jayao.2020.0013. 32392434 PMC7864108

[bibr130-13674935231208502] Pritchard MT Butow PN Stevens MM Duley JA (2006) Understanding medication adherence in pediatric acute lymphoblastic leukemia: a review. Journal of pediatric hematology/oncology 28(12): 816–823. Available at: 10.1097/01.mph.0000243666.79303.45. 17164651

[bibr31-13674935231208502] Psihogios AM Li Y Ahmed A , et al. (2021) Daily text message assessments of 6-mercaptopurine adherence and its proximal contexts in adolescents and young adults with leukemia: a pilot study. Pediatric blood & cancer. Blood & Cancer, e28767. Available at: 10.1002/pbc.28767. PMC10313157 33073479

[bibr32-13674935231208502] Pyke-Grimm KA Schulz GL Pearson H , et al. (2020) Treatment decision making. In: Hinds P Linder L (eds) Pediatric Oncology Nursing: Defining Care through Science. Springer, pp. 169–190.

[bibr33-13674935231208502] Quittner AL Espelage DL Levers-Landis C , et al. (2000) Measuring adherence to medical treatments in childhood chronic illness: considering multiple methods and sources of information. Journal of Clinical Psychology in Medical Settings 7(1): 41–54. DOI: 10.1023/A:1009545319673.

[bibr34-13674935231208502] Ruddy K Mayer E Partridge A (2009) Patient adherence and persistence with oral anticancer treatment. CA: A Cancer Journal for Clinicians 59(1): 56–66. DOI: 10.3322/caac.20004. 19147869

[bibr35-13674935231208502] Rutter M (1979) Protective factors in children’s responses to stress and disadvantage. Annals of the Academy of Medicine 8(3): 324–338. 547874

[bibr36-13674935231208502] Sawyer SM Azzopardi PS Wickremarathne D , et al. (2018) The age of adolescence. The Lancet Child & Adolescent Health 2(3): 223–228. DOI: 10.1016/S2352-4642(18)30022-1. 30169257

[bibr37-13674935231208502] Smith BA Shuchman M (2005) Problem of nonadherence in chronically ill adolescents: strategies for assessment and intervention. Current Opinion in Pediatrics 17: 613–618. DOI: 10.1097/01.mop.0000176443.26872.6e. 16160536

[bibr38-13674935231208502] Spinetta JJ Masera G Oppenheim D , et al. (2002) Refusal, non-compliance, and abandonment of treatment in children and adolescents with cancer: a report of the SIOP working committee on psychosocial issues in pediatric oncology. Medical and Pediatric Oncology 38(2): 114–117. DOI: 10.1002/mpo.1283. 11813177

[bibr39-13674935231208502] Taddeo D Egedy M Frappier J-Y (2008) Adherence to treatment in adolescents. Paediatrics and Child Health 13(1): 19–24. DOI: 10.1093/pch/13.1.19. 19119348 PMC2528818

[bibr40-13674935231208502] Tebbi CK Cummings MK Zevon MA , et al. (1986) Compliance of pediatric and adolescent cancer patients. Cancer 58: 1179–1184. DOI: 10.1002/1097-0142(19860901)58:5<1179::aid-cncr2820580534>3.0.co;2-e. 3731045

[bibr41-13674935231208502] Tebbi CK Zevon MA Richards ME , et al. (1989) Attributions of responsibility in adolescent cancer patients and their parents. Journal of Cancer Education 4(2): 135–142. DOI: 10.1080/08858198909527987. 2641329

[bibr42-13674935231208502] Tercyak KP Donze JR Prahlad S , et al. (2005) Multiple behavioural risk factors among adolescent survivors of childhood cancer in the survivor health and resilience education (SHARE) program. Pediatric Blood and Cancer 47: 825–830. DOI: 10.1002/pbc.20602. 16333821

[bibr43-13674935231208502] Vandermoris A Parsons KW Greenberg ML (2017) Adherence to treatment regimes in adolescent and young adult cancer patients. In: Bleyer A Barr R Reis L , et al. (eds) Cancer in Adolescents and Young Adults. 2nd edition. New York, NY Springer, pp. 565–581. DOI: 10.1007/978-3-319-33679-4.

[bibr44-13674935231208502] Vandermoris A Sampson L Korenblum C (2019) Promoting Adherence in Adolescents and Young Adults with Cancer to Optimize Outcomes: A Devolopmentally Oriented Narrative Review. : Pediatric Blood & Cancer, e28128. DOI: 10.1002/pbc.28128. 31886630

[bibr45-13674935231208502] Verbrugghe M Verhaeghe S Lauwaert K , et al. (2013) Determinants and associated factors influencing medication adherence and persistence to oral anticancer drugs: a systematic review. Cancer Treatment Reviews 39: 610–621. DOI: 10.1016/j.ctrv.2012.12.014. 23428230

[bibr46-13674935231208502] Warnick JL Pinto S Ding K , et al. (2020) Childhood obesity. In: Modi AC Driscoll KA (eds) Adherence and Self-Management in Pediatric Populations. Cambridge, MA: Academic, pp. 85–105. DOI: 10.1016/B978-0-12-816000-8.00004-9.

[bibr47-13674935231208502] Weaver MS Baker JN Gattuso JS , et al. (2016) “Being a good patient” during times of illness as defined by adolescent patients with cancer. Cancer 122(14): 2224–2233. DOI: 10.1002/cncr.30033. 27141846

[bibr48-13674935231208502] Wicks L Mitchel A (2010) The adolescent cancer experience: loss of control and benefit findings. European Journal of Cancer Care 19: 778–785. DOI: 10.1111/j.1365-2354.2009.01139.x. 20088922

[bibr49-13674935231208502] World Health Organization (2003) Adherence to Long-Term Therapies: Evidence for Action. Geneva: World Health Organization. https://apps.who.int/iris/handle/10665/42682

[bibr50-13674935231208502] Wu YP Yi J McClellan J , et al. (2015) Barriers and facilitators of healthy diet and exercise among adolescent and young adult cancer survivors: implications for behavioural interventions. Journal of Adolescent and Young Adult Oncology 4(4): 184–191. DOI: 10.1089/jayao.2015.0028. 26697268 PMC4684662

[bibr51-13674935231208502] Yağcı-Küpeli B Akyüz C Yalçın B , et al. (2017) Single institution experience on cancer among adolescents 15–19 years of age. Turkish Journal of Pediatrics 59: 1–5. DOI: 10.24953/turkjped.2017.01.001. 29168356

