# Supplementary material for: Facilitators and barriers to adherence to medical recommendations among adolescents with cancer: A systematic review
Source: J Child Health Care. 2023 Oct 21;29(2):501–22. doi: 10.1177/13674935231208502 (PMC12145485; doi:10.1177/13674935231208502)
Supplement: Supplemental Material - Facilitators and barriers to adherence to medical recommendations among adolescents with cancer: A systematic review [file sj-pdf-1-chc-10.1177_13674935231208502.pdf]

Supplementary material

Table S1

Search strategy: keywords and search terms for systematic review

Keyword	Search terms
Adolescence	*adol* OR teen* OR youth OR young OR pediatric OR paediatric*
Cancer	*cancer OR oncology OR neoplasm OR leukemia OR lymphoma OR tumor OR tumour*
Adherence	*adherence OR compliance OR self-management*

Figure S1

Summarised results of the included studies concerning the barriers / facilitators of adherence among adolescents with cancer
